# Specific antioxidant properties of human serum albumin

**DOI:** 10.1186/2110-5820-3-4

**Published:** 2013-02-15

**Authors:** Myriam Taverna, Anne-Lise Marie, Jean-Paul Mira, Bertrand Guidet

**Affiliations:** 1 Université Paris Sud - Faculté de Pharmacie, 92290, Châtenay-Malabry, France; 2 CNRS UMR 8612, Institut Galien Paris Sud, 92290, Châtenay-Malabry, France; 3 Assistance Publique des Hôpitaux de Paris, Groupe Hospitalier Universitaire Cochin-Broca-Hôtel Dieu, Medical Intensive Care Unit, 75014, Paris, France; 4 Université Paris Descartes, Sorbonne Paris Cité, Faculté de Médecine, 75006, Paris, France; 5 Cochin Institute, INSERM U1016/CNRS UMR 8104, 75014, Paris, France; 6 Assistance Publique des Hôpitaux de Paris, Hôpital Saint-Antoine, Medical Intensive Care Unit, 75012, Paris, France; 7 Université Pierre et Marie Curie - Paris 6, 75005, Paris, France; 8 INSERM, Unité de Recherche en Épidémiologie Systèmes d’Information et Modélisation (U707), 75012, Paris, France

**Keywords:** Human serum albumin, Antioxidant force, Oxidized albumin, Critically ill patients

## Abstract

Human serum albumin (HSA) has been used for a long time as a resuscitation fluid in critically ill patients. It is known to exert several important physiological and pharmacological functions. Among them, the antioxidant properties seem to be of paramount importance as they may be implied in the potential beneficial effects that have been observed in the critical care and hepatological settings. The specific antioxidant functions of the protein are closely related to its structure. Indeed, they are due to its multiple ligand-binding capacities and free radical-trapping properties. The HSA molecule can undergo various structural changes modifying its conformation and hence its binding properties and redox state. Such chemical modifications can occur during bioprocesses and storage conditions of the commercial HSA solutions, resulting in heterogeneous solutions for infusion. In this review, we explore the mechanisms that are responsible for the specific antioxidant properties of HSA in its native form, chemically modified forms, and commercial formulations. To conclude, we discuss the implication of this recent literature for future clinical trials using albumin as a drug and for elucidating the effects of HSA infusion in critically ill patients.

## Review

### Introduction

Clinically, Human Serum Albumin (HSA) is used for the restoration of blood volume, emergency treatment of shock, acute management of burns, and other situations associated with hypovolemia [1]. Many studies have focused on the use of albumin as a resuscitation fluid and its effects on patient outcomes in Intensive Care Units (ICU). Some of them have revealed that use of either albumin or normal saline for resuscitation fluid results in similar outcomes [2]. Others have shown negative impacts: for example, in a prospective cohort study, hyperoncotic albumin infusion was associated with harmful effects on renal function and outcome [3]. Conversely, other studies have demonstrated positive effects or beneficial trends of albumin infusion [4]. In a large controlled, randomized trial, the administration of albumin may have decreased the risk of death in patients with severe sepsis compared with saline [5]. Furthermore, a randomized, controlled trial in patients with cirrhosis and spontaneous bacterial peritonitis has shown that the addition of an albumin infusion to an antibiotic treatment significantly reduced the incidence of renal impairment and death [6]. The several meta-analyses assessing the effectiveness of albumin solutions for resuscitation in critically i1l patients failed to clarify this issue [7-10]. In this context, the choice of a resuscitation fluid in ICU patients is still a conflicting matter under debate, and it is not the scope of the present review to provide an answer to this issue. Our objective is rather to highlight the mechanisms by which albumin might exert its potential beneficial effects in the critical care and hepatological settings.

Among the hypotheses that have been proposed to explain the positive effects of albumin, its antioxidant properties seem to be of paramount importance [11-14]. This hypothesis is supported by the central role of oxidative stress in critical pathologies, such as sepsis or liver failure [1,15]. Indeed, the pathophysiology of sepsis may be characterized by the negative role of free radicals during the onset, progression, and outcome of sepsis [16,17]. Reactive Oxygen Species (ROS) and Reactive Nitrogen Species (RNS) exert their detrimental effects at least partially, through endothelial dysfunction with alterations of vascular tone, increased cell adhesion, and vascular permeability [18,19]. The activation of endothelial cells contributes to maintain the oxidant-rich environment at the inflammatory locus [20]. Moreover, high levels of ROS and RNS can result in organ damages [21], the ROS amount being related to the severity of sepsis and mortality [20]. In the same way, oxidative stress is very high in cirrhotic patients and plays an important role in the pathophysiological mechanisms involved in the hemodynamic disturbances observed [16,22]. Indeed, excessive systemic Nitric Oxide (NO) production is involved in peripheral vasoplegia, which induces portal hypertension, an important complication of cirrhosis [23]. The detrimental role of oxidative stress occurs during both the onset of hepatic alterations and during progression stages of the disease, in correlation with the severity of cirrhosis [24].

The detrimental role of oxidative damage in critical pathologies, added to the demonstration of potential positive effects of albumin infusion in these patients, constitute a strong rationale that justifies for addressing the specific antioxidant capacities of HSA [11-14]. Our review summarizes the state of the art on this topic and focuses on the antioxidant properties of HSA related to (i) first its native form, (ii) the chemically modified-HSA (iii) the commercial HSA. The implication of this recent literature for future clinical trials using albumin as a drug is finally discussed.

### Specific antioxidant capacities of native HSA

Albumin is a non-glycosylated protein of 66 kDa [25,26]; its normal plasma concentration is between 35 and 50 g/l constituting up to 60% of total plasma proteins [27]. Its half-life is ~ 20 days in normal conditions. The HSA structure consists of a single-chain polypeptide of 585 amino acid residues and approximately 67% alpha-helix and no beta-sheet [28,29]. Native HSA contains 6 methionines and 35 cysteine residues involved in the formation of 17 disulfide bonds. The Cys-34 residue is the only free cysteine in the whole molecule. HSA exerts specific antioxidant functions due to its multiple ligand-binding capacities and free radical-trapping properties, both closely related to its structure [16,29].

#### HSA antioxidant properties related to ligand-binding capacities

HSA is well known for binding a large variety of molecules, including fatty acids, drugs, hormones, and metal ions [27]. The main ligands of HSA implied in direct or indirect antioxidant functions of the protein are transition metal ions (copper and iron essentially) [15]. The high affinity site for Cu(II) ions is composed of the first four amino acids Asp-Ala-His-Lys (DAHK) from the N-terminus of HSA [30,31]. Free redox-active transition metal ions (Cu(II) and Fe(II)) can potentially be extremely pro-oxidant. Indeed, through the Fenton reaction, they can interact with hydrogen peroxide (H_2_O_2_) catalyzing the formation of aggressive ROS. The net result of the reaction sequence is known as the Haber-Weiss reaction, showing that iron and copper are the most important transition metals in human disease and play a key role in the production of hydroxyl radicals *in vivo*[30,32]. The binding of free transition metals to proteins can control their reactivity and limit their availability for the Fenton reaction [30,32]. HSA might then be able to limit damage caused by hydroxyl radicals produced from Fenton reaction between iron/copper and H_2_O_2_[31].

Other aspects of the antioxidant activity of albumin result from to its ability to bind bilirubin, homocysteine, and lipids but are of minor importance compared with metal ion involvement in HSA antioxidant properties. HSA contains one high affinity site (Lys240) for bilirubin [33]. The resulting HSA-bound bilirubin acts as an inhibitor of lipid peroxidation and thus represents an indirect antioxidant property of HSA [34]. Another aspect of HSA antioxidant properties is its capacity to bind homocysteine, a sulfur-containing amino acid resulting from the catabolism of methionine residue [35]. Some previous studies have suggested that the binding of HSA to polyunsaturated fatty acids and sterols could contribute to its antioxidant properties, by preventing lipid peroxidation. Additional studies are necessary to document this HSA protective capacity [36,37].

#### HSA antioxidant properties related to free radical-trapping properties

**Figure 1:**
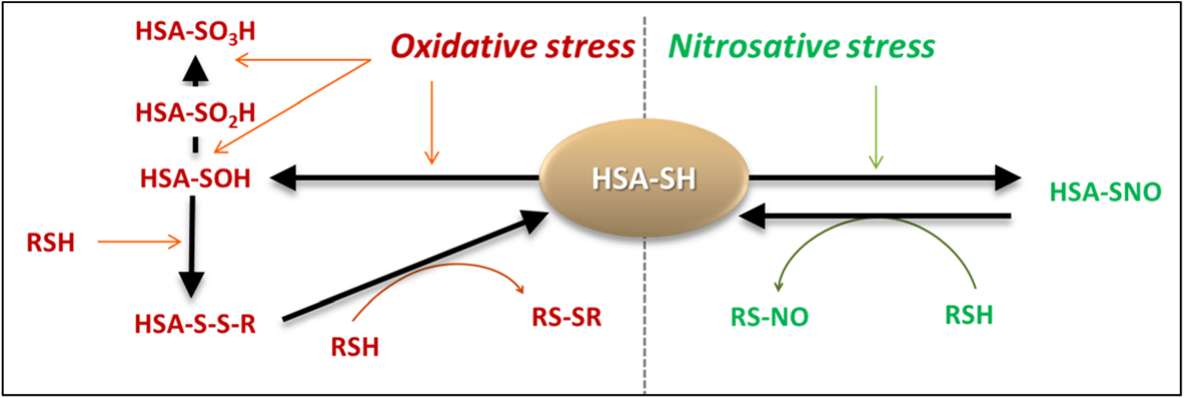
**Scheme gives an overview of the steps leading to Cys-34 oxidation and thiolation (highlighted in red). **Steps involved for the nitrosylation of Cys-34 of human serum albumin (HSA) are highlighted in green. Formation of higher oxidation states of HSA is also shown. RSH, glutathione or free cysteine.

Upon secretion in physiological conditions, one-third of the HSA pool exists as disulfides mixed with cysteine, homocysteine, or glutathione (GSH) (HSA-S-S-R) and two-thirds of the HSA molecules exist in a reduced form with a free thiol in the Cys-34 residue (HSA-SH), known as human mercaptalbumin [37,38]. This redox thiol group, in connection with the high concentration of HSA in the circulation, accounts for 80% thiols in plasma, constituting the major extracellular source of reactive free thiol [16,25,39]. Working as a free radical scavenger, the Cys34 residue is able to trap multiple ROS and RNS, such as hydrogen peroxide (H_2_O_2_), peroxynitrite (ONOO-), superoxide, or hypochlorous acid (HOCl) [15,16,40]. Under oxidative stress by peroxynitrite or hydrogen peroxide, Cys34 thiol shifts to an exposed conformation and is oxidized itself resulting in sulfenic acid (HSA-SOH) formation. HSA-SOH is a central intermediate in the redox modulation by reactive species. The final outcome of the oxidative process depends on whether the sulfenic acid is further oxidized, or whether it is reduced leading to the initial HSA-SH. Sulfenic acid may be oxidized to either sulfinic (HSA-SO_2_H) or sulfonic (HSA-SO_3_H) acids, by usually irreversible processes leading to end products [41,42]. Sulfenic acid also can be converted into a disulfide (HSA-S-S-R), through reactions with low-molecular-mass-thiol (RSH, glutathione or free cysteine) allowing the return to the HSA-SH reduced form [37,43]. This HSA implication in disulfides formation supports a relevant function of HSA-SH as an extracellular redox regulator (Figure 1) [42]. HSA also could protect cells against oxidative stress by modulating the cellular GSH level. Indeed, catabolism of HSA could potentially represent a source of sulfur-containing amino acids for cells in the synthesis of thiol-containing molecules, such as GSH [44].

Under nitrosative stress by NO or other nitrosylating agents, mercaptalbumin can be converted into nitroso-HSA (HSA-S-NO) [37]. HSA-S-NO can transfer the nitrosonium cation to low-molecular-mass-thiol RSH (glutathione or free cysteine). The HSA-mediated catalysis of RSH allows the return to the HSA-SH form and makes the protein a major reservoir of NO (Figure 1) [37,45].

As cysteine amino acid, methionine also is a sulfur-containing residue, representing an oxidation-sensitive amino acid [46,47]. HSA contains six methionines residues that can be oxidized, leading to methionine sulfoxide [48].

The two sulfur-containing residues in HSA, Met and Cys, have then been proved to account for 40-80% of the total antioxidant activity of the protein, which is responsible for more than 70% of the free radical-trapping activity in serum [28,46,47]. In conclusion, HSA is considered as the main extracellular molecule responsible for maintaining the plasma redox state [11,37,49].

### Impairments of specific antioxidant capacities of HSA

The various structural modifications that HSA can undergo during its *in vivo* lifetime or during the processes employed to isolate therapeutic HSA from plasma, modify not only its conformation and hence its binding properties but also its redox state [16,50,51]. Kawakami et al. have clearly shown that reduced HSA and oxidized HSA have different ligand-binding properties [50]. These authors also have investigated the potential effect of oxidation on the antioxidant capacity of HSA by comparing the radical scavenging activities of HSA in various states of oxidation [50]. By using electron spin resonance, they have observed that the hydroxyl radical signal is reduced from 68.7% with highly oxidized cysteinylated HSA (proportion of HSA reduced = 8%) to 54.4% with non-oxidized HSA (proportion of HSA reduced = 73%). They have then demonstrated that the radical scavenging activity of reduced HSA is greater than that of cysteinylated HSA [50]. Those results are in accordance with other reports establishing that oxidized HSA decreases scavenging ability against highly ROS (hydroxyl radicals). Similarly, HSA nitrosylation leads to a significant loss of its buffering capacity [52]. Moreover, the loss of aspartate-alanine from the N-terminus of albumin completely abolishes the ability of albumin to chelate free copper and is associated with the loss of its free radical scavenging ability [52]. Finally, Iwao et al*.* (2006) demonstrated that oxidation of several amino acids of albumin had an impact on its pharmacokinetics by decreasing its half-life [53].

Beyond oxidation and nitrosylation, HSA may undergo other chemical modifications affecting its structure, which is closely related to its specific antioxidant properties [27]. One of them is a non-enzymatic glycation consisting in the attachment of free carbohydrate (glucose, galactose, fructose…) on an amine residue leading to the formation of a stable fructosamine residue [54]. Accumulation of numerous studies has permitted to identify up to 29 sites that can be glycated *in vivo* in the HSA [34]. Due to their high nucleophile properties, lysine, arginine and cysteine are the main residues prone to glycation and the two principal sites are Arg410 and Lys525 [55,56]. Glycation-induced modifications have an important impact on HSA functional properties mainly related to alteration of its conformation. These conformational changes affect HSA binding properties because affinity of glycated HSA for different ligands (long chain fatty acids, bilirubin, copper) undergoes an important decrease [57]. Through, these various processes, the antioxidant capacities of glycated HSA are dramatically reduced [54,58,59].

In physiological conditions, due to the large amount of HSA in plasma, impairments in the protein molecule and its antioxidant properties have been considered as biologically insignificant [31]. However, antioxidant properties of modified HSA may be related to pathological conditions, in particular in septic and cirrhotic conditions in which the decline of HSA plasma antioxidant force also is mediated by the quantitative reduction of the HSA concentration [16,20,28,60].

### Specific antioxidant capacities of commercial HSA

The finding that HSA exerts a plasma antioxidant force also is important regarding its possible therapeutic effects, because it may provide the opportunity to enhance endogenous antioxidant protection in pathological conditions by HSA infusion [61]. Commercially available HSA solutions are complex products that contain not only native HSA but also various species of HSA under different redox states, as well as several minor degradation products [46,62]. Significant HSA variability has been reported between the commercial HSA solutions (Table 1). Bioprocesses and storage conditions increase heterogeneity of HSA: truncated, cysteinylated, nitrosylated, and glycosylated forms or HSA-HSA dimers may be found in commercialized products [29]. These frequently encountered modifications can change HSA antioxidant properties and its binding capacity to endogenous or exogenous molecules [29,51,63]. The observed heterogeneity also can potentially influence clinical outcome and should be correlated with morbidity/mortality in randomized trials [47,63,64]. The use of an antioxidant for the treatment of sepsis has been considered a new, interesting, adjunctive therapy and constitutes a challenge in the clinical management of these patients [17]. However, it is still unclear whether the administration of commercially available HSA that is largely oxidized, increases or decreases oxidative stress burden in critically ill patients [52].

**Table 1:** **Characteristics of commercial HSA (available solutions for infusion in France)**[29]

	**Human albumin baxter 200 g/l^®^**	**Albunorm^®^**	**Vialebex^®^**
Manufacturer	Baxter	Octapharma	LFB
Available presentations	***Newborns and infants:***		
			**20% (200 mg/ml):** 10 ml vial (2 g)
	***Adults:***		
	**20% (200 mg/ml):**	**20% (200 mg/ml):**	**20% (200 mg/ml):**
	50 ml vial (10 g)	50 ml vial (10 g)	50 ml vial (10 g)
	100 ml vial (20 g)	100 ml vial (20 g)	100 ml vial (20 g)
		**5% (50 mg/ml):**	**5% (50 mg/ml):**
		100 ml vial (5 g)	250 ml vial (12.5 g)
		250 ml vial (12.5 g)	500 ml vial (25 g)
		500 ml vial (25 g)	
		**4% (40 mg/ml):**	**4% (40 mg/ml):**
		100 ml vial (4 g)	100 ml vial (4 g)
		250 ml vial (10 g)	250 ml vial (10 g)
		500 ml vial (20 g)	500 ml vial (20 g)
Indications (EMA)	Restoration and maintenance of circulating blood volume where volume deficiency has been demonstrated and the use of a colloid is appropriate. The choice of albumin rather than an artificial colloid will depend on the clinical situation of the individual patient, based on official recommendations.		

Until now, several analytical techniques have been employed to monitor the heterogeneity or the degradation profiles of HSA. High-Performance Liquid Chromatography (HPLC), Mass Spectrometry (MS), Sodium Dodecyl Sulfate-PolyAcrylamide Gel Electrophoresis (SDS-PAGE) and Western blot analyses, or Capillary Zone Electrophoresis (CZE) have contributed to point out this heterogeneity of commercial HSA [51,62]. Aggregation or chemical degradation can be induced during sample preparation or purification. Procedures, such as temperature variations, freeze-thawing process, mechanical agitation, or lyophilization, may affect HSA structure and composition of therapeutic HSA. Anraku et al*.* studied the protective effect of sodium N-acetyl-L-tryptophanate against albumin oxidation, using HPLC. They partially resolved mercaptalbumin (reduced form of HSA) from two populations of non-mercaptalbumin (oxidized forms), demonstrating that it is possible to improve the quality of the solutions [65]. Ogasawara et al. used SDS-PAGE and Western blot analyses to detect albumin disulfide dimers in plasma, considered to be a biomarker of oxidative stress [66]. More recently, Qian et al. (2008) reported a size exclusion HPLC method to estimate the proportion of HSA dimers and oligomers suitable for a quality control [67]. MS and MS coupled to HPLC also have been employed to characterize different variants of HSA in commercial HSA preparations. In particular, six related proteins have been identified but the method provided only qualitative data and did not detect any dimer form of HSA [52]. Finally, Alahmad et al. developed a reproducible CZE method to separate HSA from most of its variants. This method proved to be useful in detecting quantitative differences in the proportion of native HSA present in batches produced according to different fractionation ways [51]. Because an increased percentage of oxidized HSA is responsible for impaired HSA functions [28], the development of a reliable method providing qualitative and quantitative data on HSA variants in commercial preparations, especially the ratio of native HSA to degraded forms, is of paramount importance for optimizing the clinical use of HSA.

### Implications for further studies

As we have previously mentioned, studies using HSA as a resuscitation fluid have shown conflicting results in septic and cirrhotic conditions. The relative contribution of resuscitation fluid capacity and antioxidant properties should be further investigated because it may influence the design of future trials. As a matter of fact, the rationale for infusing HSA was different in recent albumin trials which may account for different results. Should we rely on albuminemia and at what threshold: 30 g/l, or specific plasma concentrations (e.g. thiols, markers of ROS aggression)? The clinical studies have used different quantities of HSA infusion and various commercial forms of HSA. Regarding the HSA quality, it is well established that significant variability exists between the different commercial HSA solutions. They contain native HSA associated with various species of HSA in different redox states. They also contain several minor degradation products [47,63]. These modifications are known to change the antioxidant capacities of HSA and may account for the observed heterogeneity in the results of clinical trials [29,46,51,63,64]. It would be interesting to conduct an analysis correlating, for each clinical trial, the qualitative and quantitative characteristics of HSA infusion with the observed results in critically ill patients.

Moreover, the main characteristics of commercially available HSA should be tested *in vitro*, because they may differ from one product to another with different *in vivo* effect. The elucidation of the composition of commercialized HSA used in critically ill patients, with particular attention to the oxidized forms of HSA, is of great interest for the understanding of the observed variability in the results of clinical trials.

Finally, it is relevant to pursue the investigations concerning HSA antioxidant functions in physiological and pathophysiological conditions. Accumulative evidence suggests that HSA may interfere with microcirculation and endothelium function through an interaction with glycocalyx and specific antioxidant properties [68]. As a consequence, monitoring of endothelial function and microcirculation may guide HSA administration and contribute to the optimization of tissue perfusion in septic shock. The preliminary results of the Albios trial (presented orally at the ESICM meeting in Lisbon October 2012) suggest that HSA was effective in the subgroup of patients with the most severe form of sepsis which is septic shock. Future trials should probably focus on that specific high-risk population [69].

## Conclusions

It is well established that, among plasma antioxidants, endogenous HSA is considered the main extracellular molecule responsible for maintaining the plasma redox state [16,29]. Its specific antioxidant functions are due to its multiple ligand-binding capacities and free radical-trapping properties and are closely related to the structure and the redox state of the molecule [16,29]. Some clinical studies have revealed positive effects or beneficial trends of HSA infusion [4,5]. Importantly, these trials have been conducted in critical pathologies in which oxidative stress plays a central detrimental role [1,15]. It seems relevant to consider that the specific antioxidant properties of the HSA molecule are involved in the positive therapeutic effects of HSA infusion, reported in the critical care and hepatological setting. In this hypothesis, using HSA as a resuscitation fluid could represent an opportunity to enhance endogenous antioxidant protection in critical pathological conditions [1,61]. Because an increased percentage of oxidized HSA is responsible for impaired HSA functions [50], we propose that preference should be given to preparations with a higher reduced HSA percentage.

## Abbreviations

Cys: Cysteine; CZE: Capillary Zone Electrophoresis; GSH: Glutathione; HPLC: High-Performance Liquid Chromatography; HSA: Human Serum Albumin; ICU: Intensive Care Units; Met: Methionine; MS: Mass Spectrometry; NO: Nitric Oxide; RNS: Reactive Nitrogen Species; ROS: Reactive Oxygen Species; SDS-PAGE: Sodium Dodecyl Sulfate-PolyAcrylamide Gel Electrophoresis.

## Competing interests

Pr Myriam Taverna, Anne-Lise Marie, and Pr Bertrand Guidet have received honorarium from LFB Biomedicaments and Fresenius Kabi. Pr Jean-Paul Mira has received honorarium from LFB Biomedicaments and Baxter.

## Authors’ contributions

All authors contributed to the drafting of the manuscript and approved the final version.
